# Construction of a novel 3-gene diagnostic signature related to senescence in intervertebral disc degeneration

**DOI:** 10.1186/s40001-025-03424-3

**Published:** 2025-11-26

**Authors:** Haoxi Li, Mingke Wei, Chengqiang Yu, Zhuhai Li, Qie Fan, Shuyu Yao, Yufeng Huang, Jianxun Wei

**Affiliations:** 1 Department of Spine Surgery, The People’s Hospital of Guangxi Zhuang Autonomous Region, Guangxi Academy of Medical Sciences, Nanning, 530016 China; 2 Department of Spine Surgery, Shanghai East Hospital, School of Medicine, Tongji University, Shanghai, 200092 China

**Keywords:** Senescence, Machine learning, Intervertebral disc degeneration, Biomarkers, Diagnostic model, Immune landscape

## Abstract

**Background:**

Numerous studies have manifested that cellular senescence involves in the pathogenesis of intervertebral disc degeneration (IDD). Here, we constructed a novel senescence-related genes (SRGs) signature for IDD.

**Methods:**

Three data sets were derived from Gene Expression Omnibus (GEO) database and 370 SRGs were collected from cellAge database. Key module genes related to senescence in IDD were screened using “WGCNA” package. The “limma” package was employed to filter differentially expressed genes (DEGs) between control and IDD groups, and candidate genes were identified by intersecting up-regulated DEGs and module genes. Hub genes were screened by randomForest and LASSO regression analysis. Diagnosis performance of hub genes was assessed and verified by receiver operating characteristic (ROC) curve. Diagnosis biomarkers of IDD were identified based on AUC > 0.7. Signaling pathway enrichment analysis of biomarkers was performed using “clusterProfiler” package. Immune cells infiltration was evaluated by “MCP-counter” and “GSVA” packages.

**Results:**

A total of 625 module genes and 384 DEGs were obtained, then 28 candidate genes related to senescence in IDD were screened. By machine learning, 4 hub genes were identified with good diagnostic performance, which were highly expressed in IDD samples. Furthermore, 3 biomarkers (*BID*, *KANK2*, *and SMIM3*) were screened with AUC > 0.7 in external datasets. Three biomarkers were mainly involved in nuclear factor (NF)-kappa B, TNF, IL-17, and NOD-like receptor signaling pathway, etc. Besides, *BID*, *KANK2*, and *SMIM3* exhibited positive association with most immune cell infiltration.

**Conclusions:**

We identified 3 diagnostic biomarkers related to senescence in IDD, hoping to improve the treatment of IDD.

## Introduction

Lower back pain (LBP) is a main cause of disability for patients influencing about 637 million people globally [1]. Intervertebral disc degeneration (IDD) is known as a complication of LBP, and approximately 75–85% of elderly people all around the world suffer from LBP ascribed by IDD [2]. IDD belongs to a chronic development disease with multiple molecular variant characteristics, such as the loss of nucleus pulposus cells (NPCs), abnormal apoptosis and senescence of disc cells, and degradation of extracellular matrix (ECM) [3]. Numerous factors including genetics, aging, excessive mechanical stress, disc trauma, and inflammatory reactions are associated with the occurrence of IDD [4]. However, the pathogenesis of IDD has not been comprehensively elucidated. Until now, the diagnosis of IDD is only based on clinical observation and magnetic resonance imaging (MRI) [5], and the interventions for IDD chiefly comprise conservative and surgical strategies [6], but the treatment outcomes are still unsatisfactory [7]. Early diagnosis and timely therapy could efficaciously postpone IDD progression and notably decrease the incidence of handicap [8]. Therefore, identifying effective diagnostic biomarkers and expounding its underlying pathogenesis is particularly crucial for eradicating IDD.

More and more evidences have manifested that the phenotypic change of intervertebral disc cells towards a senescent phenotype could be a main driving force of age-relevant IDD [9]. Cellular senescence is an evolutionarily conserved cell fate that can induce the accumulation of unrepaired cell damage and inconvertible cell cycle arrest, thereby resulting in the loss of cell proliferation ability [10]. Senescent cells can inappropriately release ECM proteases, pro-inflammatory cytokines (e.g., TNF-α, IL-8, IL-6, and IL-7), chemokines, and growth factors, leading to immune dysfunction or inflammatory reactions and deteriorating the clinical presentation of IDD [11]. The release of these signaling molecules is associated with the senescence-associated secretory phenotype (SASP). As a central mechanism by which senescent cells drive chronic inflammation, ECM degradation, and immune cell recruitment in degenerative diseases, the hypersecretory state of SASP affects cellular senescence [12]. The chronic accumulation of senescent cells is considered to be associated with IDD in the aging population, and their number increases in aged and degenerated intervertebral discs [10]. Studies have confirmed that cellular senescence can serve as a diagnostic marker to combat age-related IDD [9], and improving cellular senescence helps revitalize intervertebral discs [13]. Despite a good link has been established between senescence and IDD, the pathogenic mechanism of IDD contributed by cellular senescence is still unclear [14]. Thus, further exploring the diagnostic value of SRGs in IDD and their relationship with immune cell infiltration may provide novel diagnostic markers and approaches for IDD.

In recent years, biomarkers of IDD have been increasingly discovered by bioinformatics analyses, uncovering several new insights [15, 16]. Xu et al. created a diagnostic model based on 20 differential ageing-related genes in peripheral blood for IDD by machine learning [17]. Deng et al. screened 12 critical senescence-related DEGs in IDD through LASSO regression analysis that could be applied to distinguish high-risk from low-risk IDD patients, and nomogram was constructed using 4 senescence-related DEGs with AUC > 0.75 to predict the incidence rate of IDD [17]. However, the former study mainly focused on the biological functions in peripheral blood, while the latter had shortcomings including a small data set size, a limited SRGs database, and no involvement in weighted gene co-expression analysis. Based on this, to explore whether SRGs can serve as diagnostic markers for IDD, a new experiment was designed in this study. First, three datasets (GSE70362, GSE15227, and GSE147383) were acquired from the GEO database, and 370 SRGs were collected from the cellAge database. Then, by weighted gene correlation network analysis (WGCNA), DEG analysis, and two machine learning methods, hub genes of IDD were screened in GSE70362 data set. Moreover, signaling pathways involved in biomarkers and its correlation with immune cell infiltration were conducted. This study could provide promising diagnostic markers for diagnosing IDD and its management.

## Materials and methods

### Data acquisition and processing

The chip data of three datasets, GSE70362, GSE15227, and GSE147383, were collected from the GEO database (https://www.ncbi.nlm.nih.gov/geo/) [18]. Subsequently, the probes were converted to Gene Symbol based on the annotation file, removing the one probe that corresponded to multiple genes. Finally, 14 control and 10 IDD samples in GSE70362, 12 control and 3 IDD samples in GSE15227, 4 control and 4 IDD samples in GSE147383 were obtained for further analysis.

A total of 370 SRGs were acquired from the cellAge database (https://genomics.senescence.info/cells/) [19]. CellAge is a manually curated database whose gene sets are derived from genes that have been experimentally validated to be associated with the cellular senescence process in various human cell types through genetic manipulations (such as knockout or overexpression). These genes may serve as causal drivers or biomarkers of senescence and are widely used in systems biology analyses.

### WGCNA

To identify module genes significantly associated with clinical traits from the perspective of co-expression, WGCNA was conducted on the samples in GSE70362 data set using the “WGCNA” R package [3, 20], screening the key gene module related to IDD and cellAge score. The cellAge score of each sample was calculated using the “gene set variation analysis (GSVA)” R package [21]. The optimal soft threshold (β) was determined using the pickSoftThreshold function to ensure that the network was a scale-free network. Then, the co-expressed gene modules were obtained through the hierarchical clustering, with the screening criteria of minModulus Size = 200. Next, the module–trait relationships between cellAge score, IDD, and gene modules were analyzed, collecting the key gene module with highest correlation coefficient for subsequent analysis.

### DEGs analysis

The DEGs between control and IDD samples in GSE70362 dataset were analyzed using the “limma” R package [22], with a screening threshold of *p* < 0.05 and |log_2_ foldchange (FC)|> log_2_(1.5). Then, the up-regulated DEGs were intersected with the key module genes in WGCNA, obtaining the candidate genes. Meanwhile, gene ontology (GO) enrichment analysis was performed on the candidate genes by z-score using the DAVID (https://david.ncifcrf.gov/tools.jsp) online tool [23, 24].

### Identification of hub genes

The hub genes related to senescence in IDD were screened from the candidate genes by two machine learning algorithms. First of all, the randomForest model (non-linear modeling to evaluate feature importance) was constructed using the “randomForest” R package [25]. The variable number of binary tree (mtry) was determined based on the mean misjudgment rate of Outofbag (OOB) samples, and the number of decision tree (ntree) was determined based on the model error. The top 10 genes of variable importance were displayed according to MeanrecoaseAccuracy and MeanrecoaseGini, respectively. Furthermore, LASSO regression analysis (linear feature selection to avoid overfitting) was performed by tenfold cross validation using the “glmnet” R package [26], with the parameters of nfolds = 10 and family = ‘binomial’. The λ corresponding to the minimum binomialDeviance was selected as the result of LASSO. Then, the hub genes were acquired by intersecting the results of randomForest and LASSO.

### Verification of hub genes

The diagnosis performance of hub genes in IDD was assessed in GSE70362 data set by ROC curve using the “pROC” R package [27]. The AUC of each hub gene was calculated, and the expressions of hub genes between the control and IDD groups were compared. Meanwhile, the diagnosis performance of hub genes was validated in two external datasets, GSE15227 and GSE147383. The genes with AUC > 0.7 were selected as diagnostic biomarkers for IDD in this study.

### Gene set enrichment analysis

Kyoto encyclopedia of genes and genomes (KEGG) enrichment analysis was performed by the gseKEGG function using the “clusterProfiler” R package [28], and *p* < 0.05 was set as enrichment significance threshold. Then, the top 15 metabolic pathways with the highest absolute value of normalized enrichment score (NES) were selected and visualized.

### Analysis of immune cells infiltration

The infiltration levels of 10 immune cells between control and IDD groups in GSE70362 data set were compared by the “microenvironment cell populations-counter (MCP-counter)” R package [29]. The infiltration levels of 28 immune cells were evaluated by ssGSEA with the “GSVA” R package. In addition, the correlation between 28 immune cells infiltration and biomarkers was analyzed.

### Statistical analysis

The statistical analysis was performed using a R software (version 3.6.0). Wilcoxon rank sum test was utilized to compare the difference between two continuous variables. Spearman method was utilized for correlation analysis. The *p* < 0.05 stood for statistically significant.

## Results

### Magenta module exhibited the strongest correlation with cellAge score and IDD

**Fig. 1:**
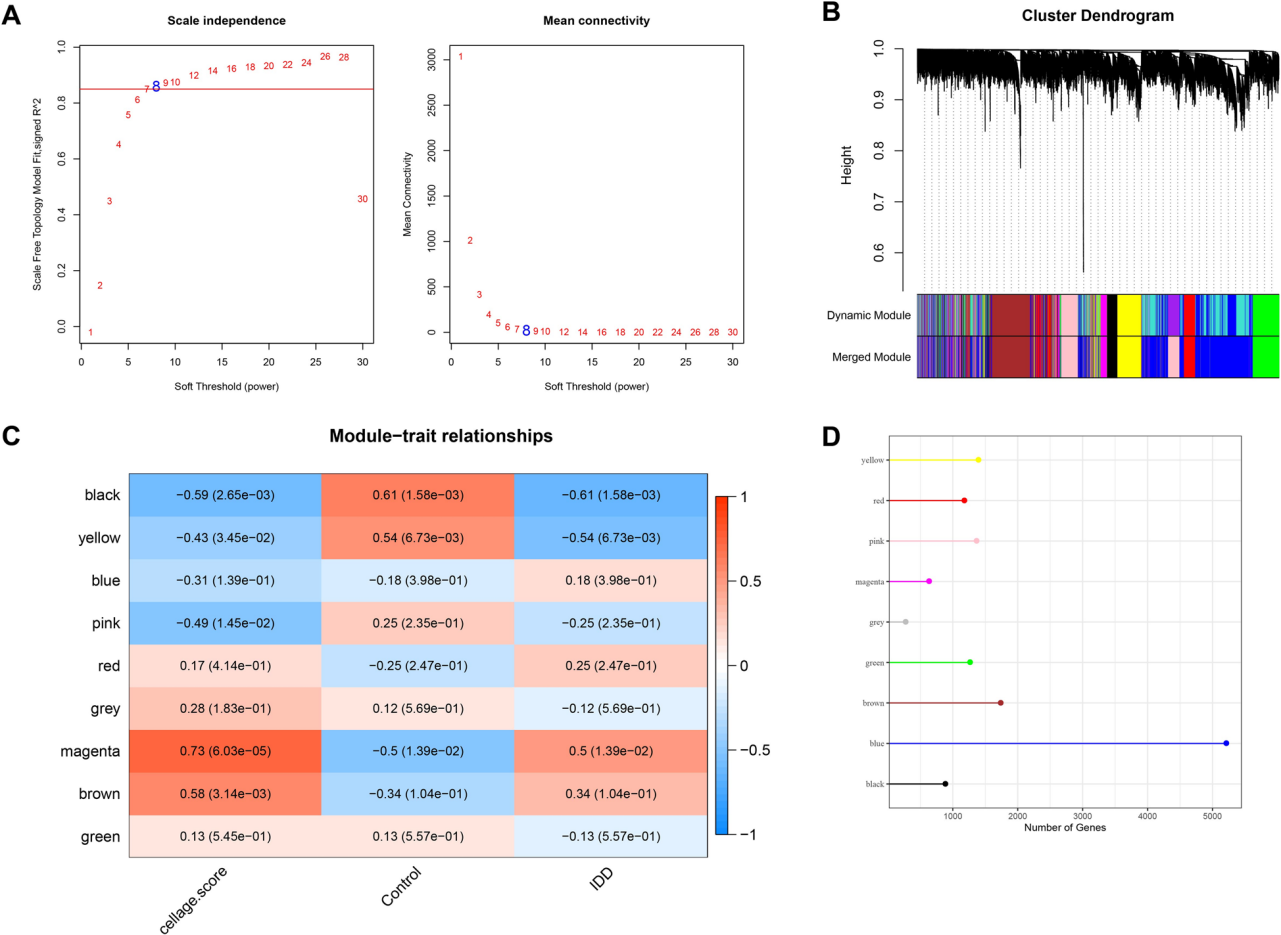
Identification of key module genes in intervertebral disc degeneration (IDD) by WGCNA. **A** Screening of the optimal soft threshold to construct scale-free topology network; **B** cluster dendrogram based on 1 topological overlap matrix (1-TOM); **C** module–trait relationships heatmap between cellAge score, IDD, and modules; **D** number of genes in each module.

The optimal soft threshold (β) was selected as 8 to construct the scale-free topology network (Fig. 1A). A total of 9 co-expressed gene modules were obtained by hierarchical clustering (Fig. 1B), and the grey module could not be aggregated to other modules. The module–trait relationship heatmap showed that magenta module had the highest correlation coefficients with cellAge score (cor = 0.73) and IDD (cor = 0.5) (Fig. 1C). Besides, the gene number of each module was displayed by a lollipop plot (Fig. 1D), and the magenta module contained 625 genes. Thus, the magenta module genes with the strongest correlation to cellAge score and IDD were selected for subsequent analysis.

### 28 candidate genes related to senescence in IDD were screened

**Fig. 2:**
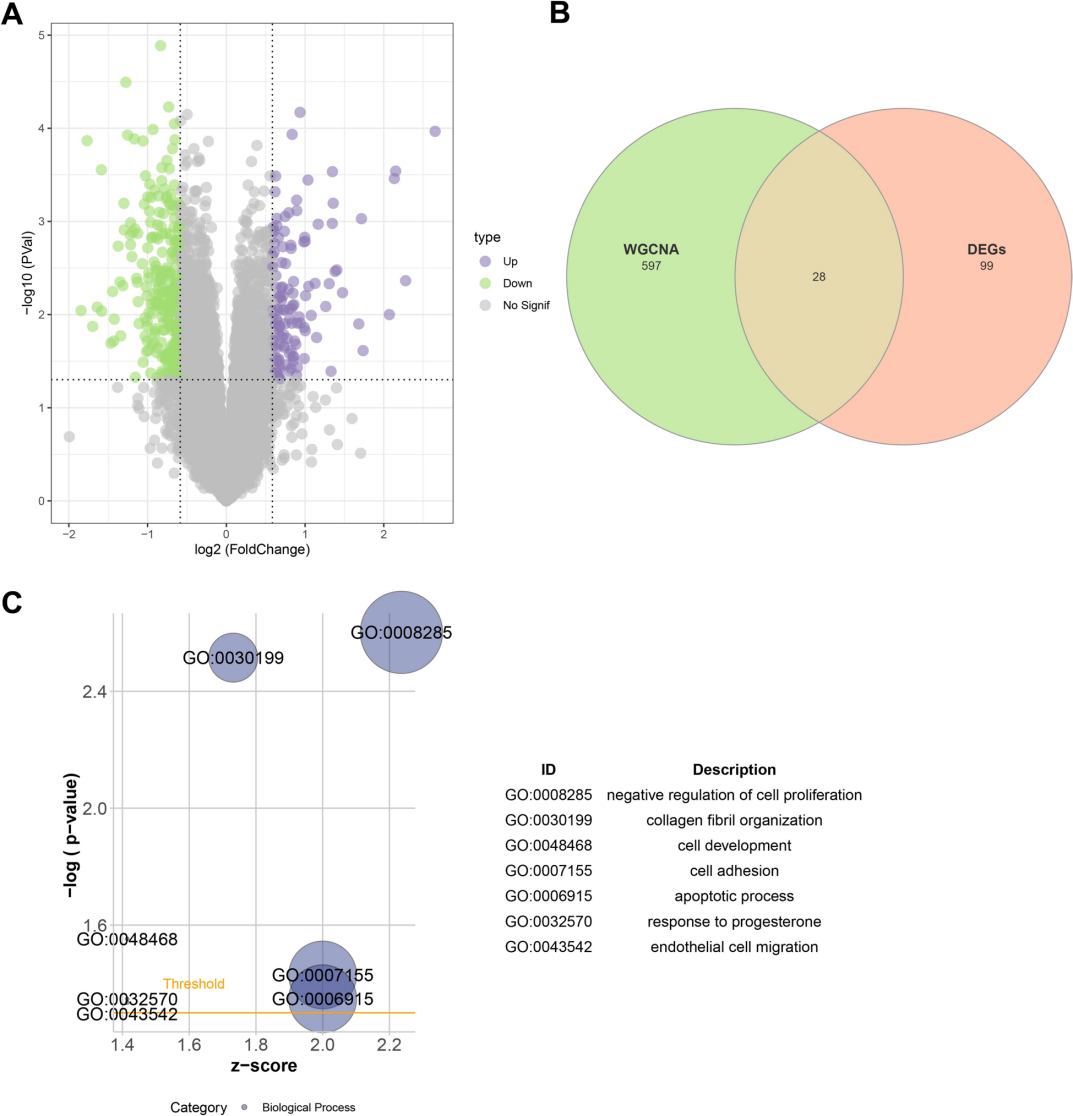
Screening of candidate genes related to senescence in IDD. **A** Volcano plot of the DEG in GSE70362 dataset; **B** Venn diagram of up-regulated DEGs and module genes; **C** Gene ontology (GO) enrichment results of candidate genes.

A total of 384 DEGs between control and IDD samples in GSE70362 dataset were obtained, including 257 down-regulated and 127 up-regulated DEGs (Fig. 2A). By intersecting the 625 magenta module genes with the 127 up-regulated DEGs, 28 candidate genes related to senescence in IDD were screened (Fig. 2B). Moreover, GO enrichment analysis in biological process suggested that the candidate genes mainly enriched in the negative regulation of cell proliferation, collagen fibril organization, cell development, cell adhesion, apoptotic process, response to progesterone, and endothelial cell migration (Fig. 2C). These biological processes may be involved in the IDD progression.

### Four hub genes associated with senescence in IDD were identified

**Fig. 3:**
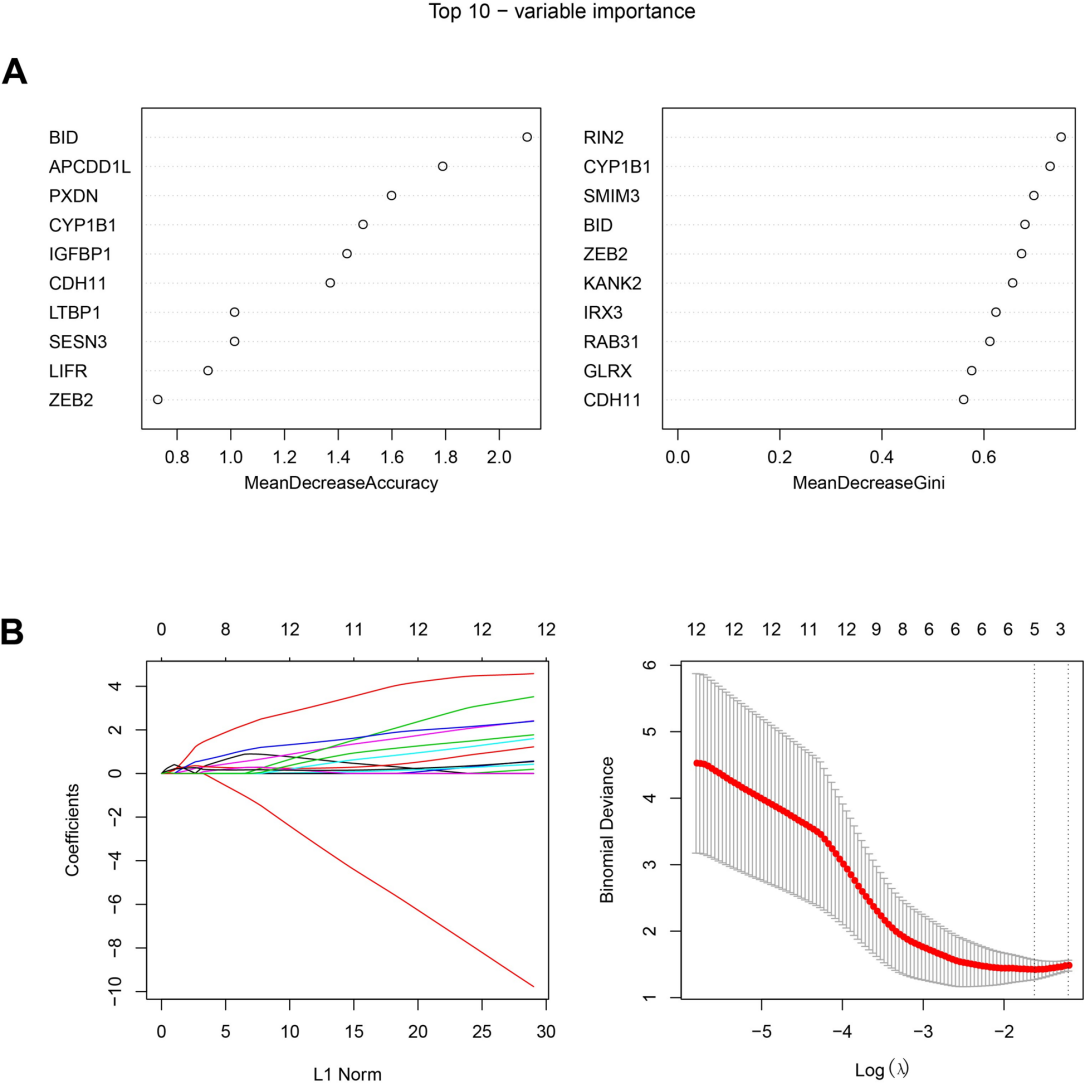
Identification of critical genes in IDD by machine learning. **A** Top 10 genes of variable importance according to MeanrecoaseAccuracy and MeanrecoaseGini by randomForest; **B** The coefficients and binomial deviance by LASSO regression analysis.

The top 10 genes of variable importance were screened by randomForest algorithm according to MeanrecoaseAccuracy and MeanrecoaseGini (Fig. 3A), respectively. By LASSO regression analysis, the λ was determined to be 5 with the minimum binomialDeviance (Fig. 3B).

**Fig. 4:**
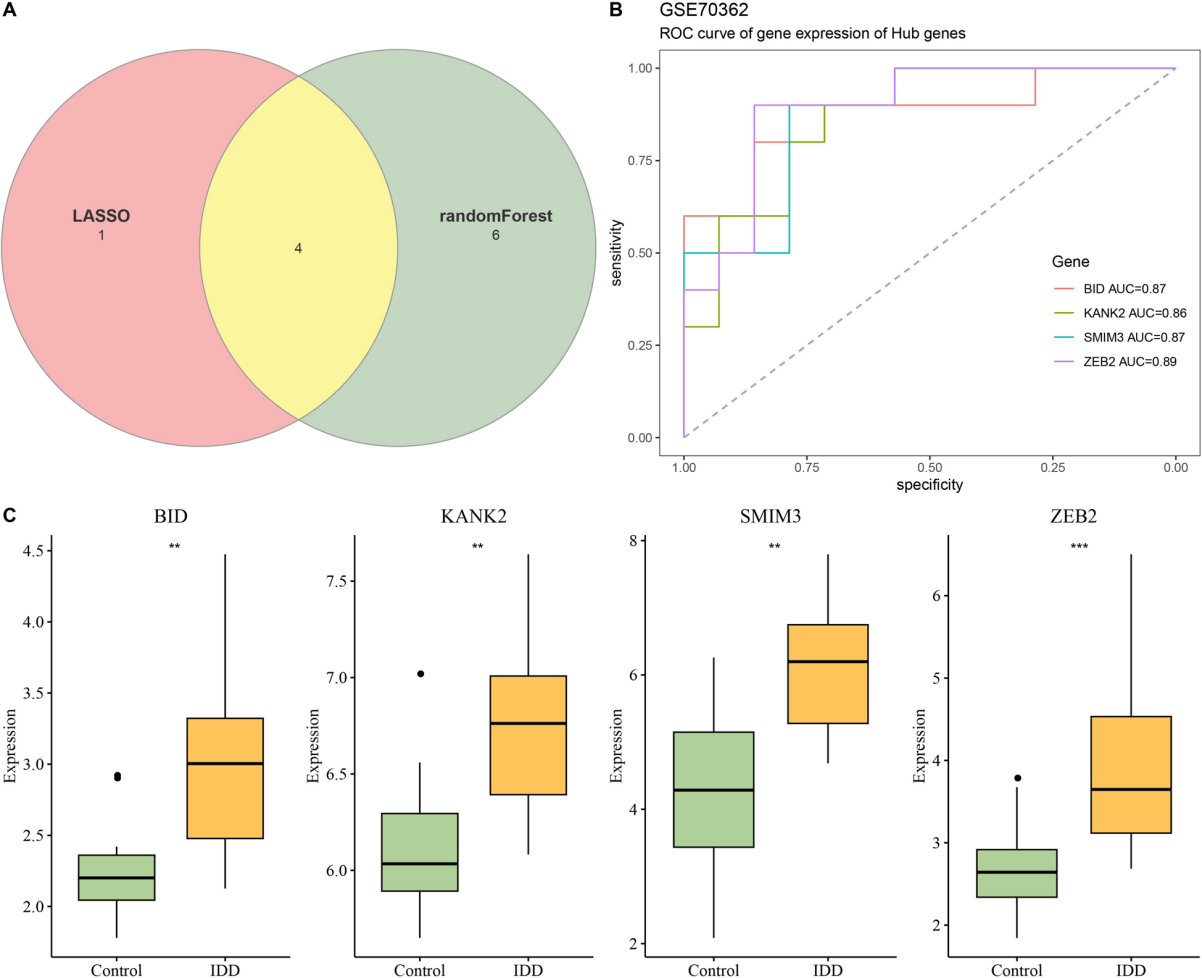
Screening of hub genes associated with senescence in IDD. **A** Venn diagram of randomForest and LASSO results. **B** ROC curves of 4 hub genes in GSE70362 dataset; **C** The expressions of 4 hub genes in control and IDD groups. ^***^ Means *p* < 0.001; ^**^ Means *p* < 0.01.

Furthermore, 4 hub genes associated with senescence in IDD were screened by intersecting the results of LASSO and randomForest algorithms (Fig. 4A), comprising *BID*, *KANK2*, *SMIM3*, and *ZEB2*. The diagnostic performance of 4 hub genes for IDD in GSE70362 dataset was evaluated by ROC curve, and the hub genes showed higher specificity and sensitivity, with *BID* AUC = 0.87, *KANK2* AUC = 0.86, *SMIM3* AUC = 0.87, and *ZEB2* AUC = 0.89 (Fig. 4B). In addition, 4 hub genes were all highly expressed in IDD samples compared to control samples (Fig. 4C).

### Three diagnostic biomarkers in IDD were identified

**Fig. 5:**
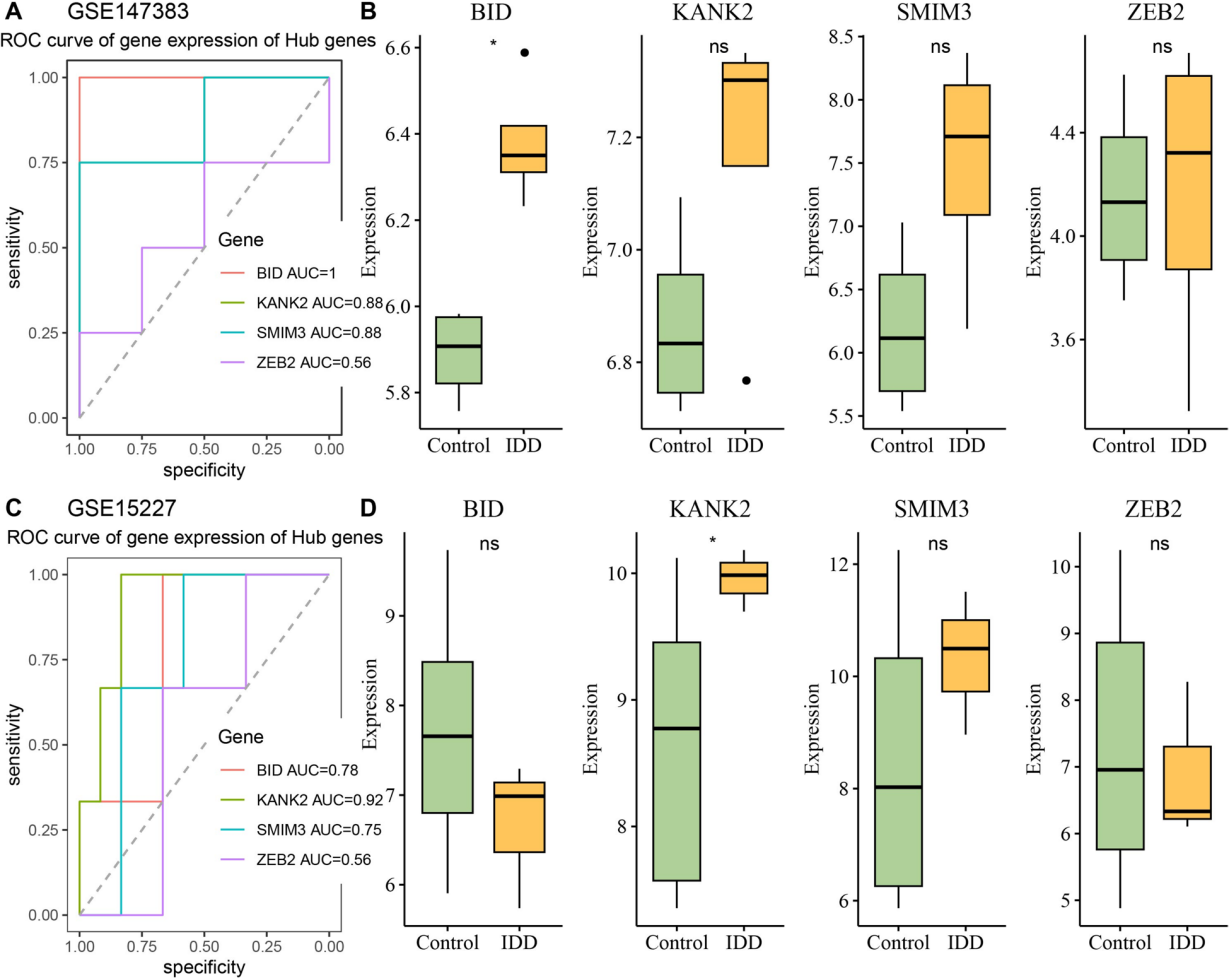
Verification of diagnostic performance of 4 hub genes in external data sets. **A** ROC curves of 4 hub genes in GSE147383 dataset; **B** The expression levels of 4 hub genes in GSE147383 dataset; **C** ROC curves of 4 hub genes in GSE15227 dataset; **D** The expression levels of 4 hub genes in GSE15227 dataset. ^*^ Indicates *p* < 0.05; ns Indicates not significant

The diagnostic performance of hub genes was verified in two external data sets. In the GSE147383 dataset, ROC curve showed that the AUC values of *BID*, *KANK2*, *SMIM3*, and *ZEB2* were 1, 0.88, 0.88, and 0.56 (Fig. 5A), respectively. The expression level of *BID* in IDD group was significantly higher than that in control group (Fig. 5B). In the GSE15227 dataset, ROC curve suggested that the AUC values of *BID*, *KANK2*, *SMIM3*, and *ZEB2* were 0.78, 0.92, 0.75, and 0.56 (Fig. 5C), respectively. Compared with control group, *KANK2* was highly expressed in IDD group (Fig. 5D). The AUC values of *BID*, *KANK2*, and *SMIM3* were all greater than 0.7 with good diagnostic performance, which were identified as the biomarkers for IDD.

### The signaling pathways relevant to biomarkers were analyzed by GSEA

**Fig. 6:**
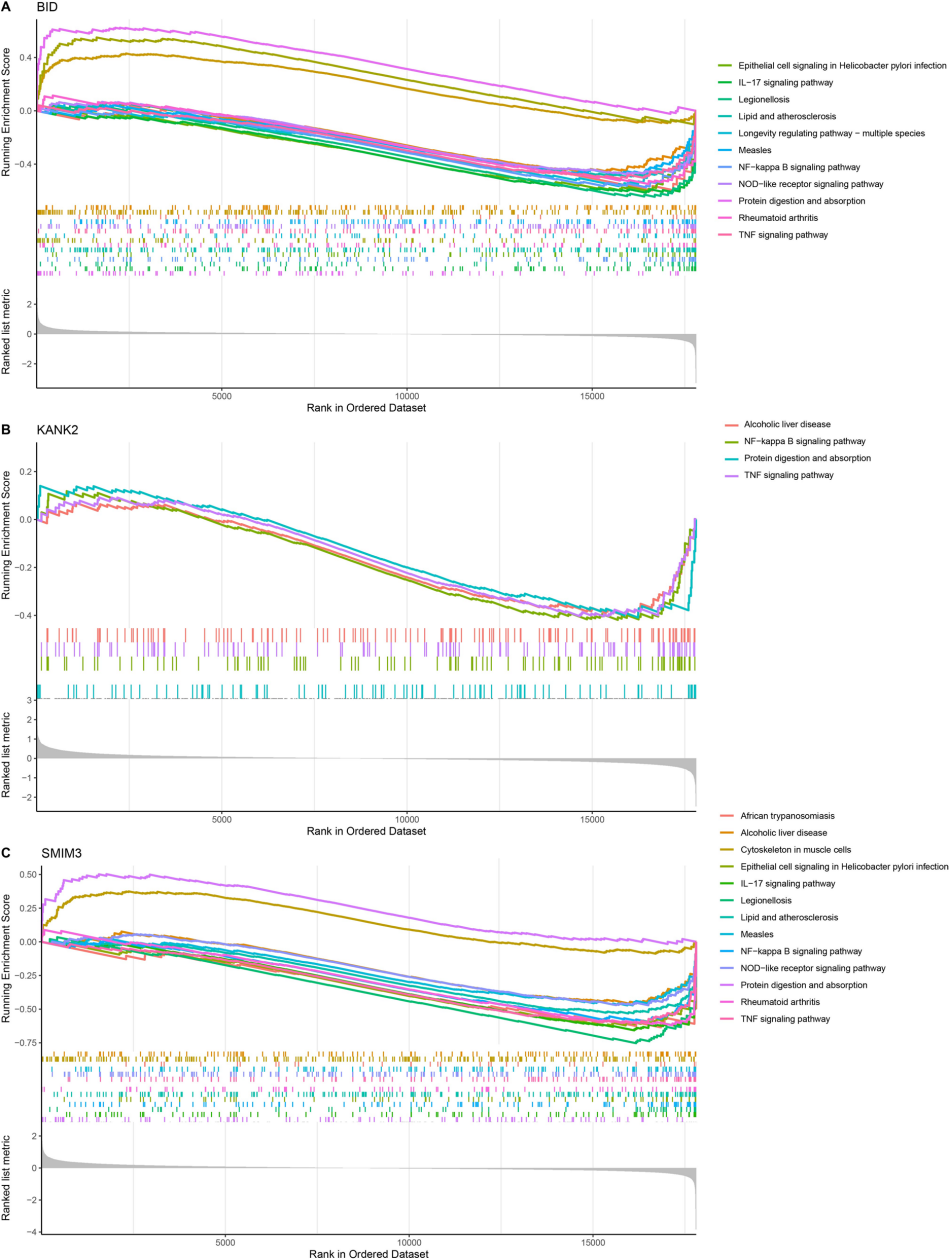
Analysis of signaling pathways relevant to biomarkers in IDD by GSEA. **A** KEGG enrichment result of *BID*; **B** KEGG enrichment result of *KANK2*; **C** KEGG enrichment result of *SMIM3*

GSEA indicated that *BID* was primarily enriched in the IL-17 signaling pathway, Longevity regulating pathway-multiple species, NF-kappa B signaling pathway, Protein digestion and absorption, NOD-like receptor signaling pathway, TNF signaling pathway, etc. (Fig. 6A). *KANK2* was principally participated in the Alcoholic liver disease, Protein digestion and absorption, NF-kappa B signaling pathway, TNF signaling pathway (Fig. 6B). *SMIM3* was chiefly involved in the Cytoskeleton in muscle cells, IL-17 signaling pathway, NF-kappa B signaling pathway, NOD-like receptor signaling pathway, Protein digestion and absorption, TNF signaling pathway, and so on (Fig. 6C). These signaling pathways relevant to the diagnostic biomarkers played a critical role in IDD progression.

### Immune cells infiltration was analyzed by MCP-counter and ssGSEA

**Fig. 7:**
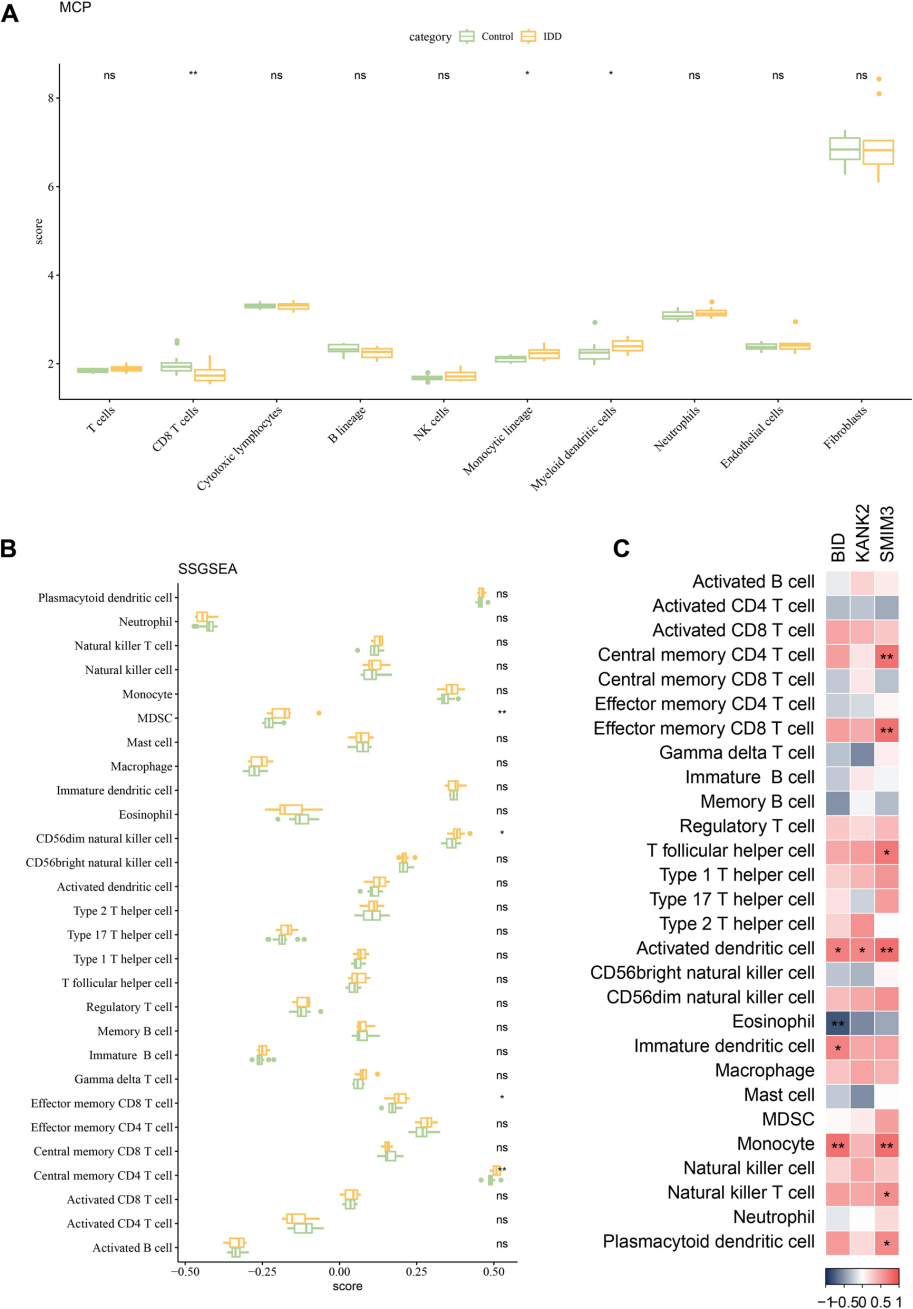
Immune cell infiltration in GSE70362 dataset. **A** Infiltration of 10 types of immune cells between control and IDD groups calculated by MCP-counter; **B** infiltration of 28 types of immune cells in control and IDD groups evaluated by ssGSEA; **C** correlation between 3 biomarkers and 28 types of immune cells infiltration by spearman method. ^**^ Means *p* < 0.01; ^*^ Means *p* < 0.05; ns Means not significant

The infiltration levels of immune cells between control and IDD samples were compared in the GSE70362 data set. MCP-counter analysis showed that compared with control, the IDD patients had higher monocytic lineage and myeloid dendritic cells infiltration, together with lower CD8 T cells infiltration (Fig. 7A). According to ssGSEA, the infiltration levels of myeloid-derived suppressor cell (MDSC), Effector memory CD8 T cell, CD56dim natural killer cell, and Central memory CD4 T cell in IDD samples were notably higher than that in control group (Fig. 7B). Furthermore, *BID* was positively correlated with the infiltration levels of Immature dendritic cell, Monocyte, Activated dendritic cell; *KANK2* was positively associated with Activated dendritic cell infiltration; *SMIM3* was positively connected with the infiltration levels of Natural killer T cell, T follicular helper cell, Plasmacytoid dendritic cell, Monocyte, Central memory CD4 T cell, Activated dendritic cell, Effector memory CD8 T cell (Fig. 7C).

## Discussion

IDD is considered as a main cause of LBP, and multiple researches have suggested that cellular senescence and SRGs exert an essential role in the progression of IDD [30]. Xu et al. created a diagnostic model based on 20 differential ageing-related genes in peripheral blood for IDD by machine learning [17]. Deng et al. screened 12 critical senescence-related DEGs in IDD through LASSO regression analysis that could be applied to distinguish high-risk from low-risk IDD patients, and nomogram was constructed using four senescence-related DEGs with AUC > 0.75 to predict the incidence rate of IDD [17]. In this current study, we found that magenta module in WGCNA exhibited the strongest correlation with cellAge score and IDD, and screened 384 DEGs between control and IDD groups. Furthermore, 4 hub genes were identified by randomForest and LASSO regression analysis, and 3 diagnostic biomarkers (*BID*, *KANK2*, *SMIM3*) with AUC > 0.7 were selected in two external datasets. *BID*, *KANK2*, and *SMIM3* were mainly involved in NF-kappa B, TNF, IL-17, NOD-like receptor signaling pathway, and exhibited positive correlation with most immune cell infiltration. Hence, constructing a diagnostic model for IDD patients based on SRGs is reliable, providing novel insights into the pathogenesis of IDD and its therapies.

*BID*, BH3 interacting domain death agonist, belongs to a pro-apoptotic member of Bcl-2 protein family and can regulate apoptosis [31]. *BID* also possesses the functions in controlling non-apoptotic cell death and mitochondrial energetics [32]. The single nucleotide polymorphism of *BID* was reported to be a risk factor and associated with ossification of the posterior longitudinal ligament in Korean patients [33]. In previous studies, BID has also been identified as a biomarker for IDD [34]. High *BID* expression disrupts the local inflammatory response of intervertebral disc cells, thereby inhibiting the anti-inflammatory effect of Bcl-2 and ultimately leading to the development of IDD [35]. *KANK2*, KN motif and ankyrin repeat domains 2, which is characterized by an N-terminal KN motif, coiled-coil domains, and a C-terminal ankyrin repeat domain [36]. *KANK2* plays an important role as a scaffold in cortical microtubule stabilization complexes, which has been manifested to serve as a promising diagnostic marker for neurodegenerative diseases by cross-species validations [37]. Studies have found that epithelial–mesenchymal transition (EMT) and angiogenesis are abnormal in cancers with high *KANK2* expression, while ECM imbalance, excessive innervation of sensory nerves and vascularization are common phenomena in IDD [38]. These findings suggest that *KANK2* may influence disease occurrence through ECM remodeling and angiogenesis [39]. *SMIM3*, located on chromosome 5q33.1, is expressed in many human organs, such as heart, ovarian, and skeletal muscle, and exerts a critical role in the regulation of cell channel and neuronal differentiation [40]. Previous studies have identified *SMIM3* as a biomarker for predicting the pain of IDD [41]. In our present study, we found that the expression levels of *BID*, *KANK2*, and *SMIM3* were remarkably elevated in IDD samples compared with control samples. These findings indicated that SRGs could serve as promising diagnostic biomarkers in IDD. Although previous studies have also identified *MAPK8* and *CAPN1* as biomarkers for IDD [42], *KANK2*, discovered in this study, is more suitable for blood testing, as stable mRNA and protein fragments of *KANK2* are detectable in the blood. Furthermore, *KANK2* is associated with fissure progression and vascularization risk in imaging evaluations [39] and holds potential as an early biomarker, which can thereby improve the early diagnosis rate of IDD.

Furthermore, GSEA showed that *BID*, *KANK2*, and *SMIM3* were primarily involved in TNF signaling pathway, NOD-like receptor signaling pathway, NF-kappa B signaling pathway, and IL-17 signaling pathway, suggesting that the inflammatory response may be a crucial pathological process in IDD [43]. To date, multiple studies have manifested that some inflammatory factors and pathways are involved in the occurrence and development of IDD [44]. For instance, the activation of NF-kappa B signaling pathway could enhance the expressions of numerous inflammatory mediators or chemokines, resulting in the malignant progression of IDD [45]. TNF-α was reported to be upregulated in IDD and correlated with many pathological processes (such as inflammation, oxidative stress, cellular senescence, apoptosis), and suppression of TNF-α signaling pathway could attenuate the IDD development [42]. The IL-17 expression level exhibited positive correlation with the severity of IDD, which could facilitate the progression of IDD via adjusting inflammatory response, ECM metabolism, and angiogenesis [46]. In addition, immunoreaction is a vital driving factor of inflammation, and a variety of immune cells participate in this pain-inducing process of IDD [47]. In this study, we found that *BID*, *KANK2*, and *SMIM3* were positively connected with most immune cell infiltration, including Central memory CD4 T cell, Effector memory CD8 T cell, T follicular helper cell, Activated dendritic cell, Monocyte, and Natural killer T cell. Some of these immune cells could release abundant pro-inflammatory molecules, promoting the inflammatory cascade reaction within the intervertebral disc [48]. Notably, *BID*, *KANK2*, and *SMIM3* all exhibit a significant positive correlation with activated dendritic cells. As a specialized and heterogeneous population of antigen-presenting innate immune cells, dendritic cells, upon activation, produce various cytokines and chemokines, uptake and process antigens [49], and can induce both immune activation and immune tolerance [50]. Furthermore, dendritic cells serve as a key bridge connecting innate immunity and adaptive immunity, by recognizing pathogen signals, they further shape the body’s acute inflammatory response, thereby may playing a central role in immune defense and inflammatory regulation of IDD [51]. Therefore, *BID*, *KANK2*, and *SMIM3* may be involved in the progression of IDD by regulating inflammatory signaling pathways and immune cell infiltration, though the specific mechanisms require further investigation.

There are still several shortcomings that deserve our attention. First of all, the sample size of the GSE70362, GSE15227, and GSE147383 datasets used in this study is so small that it may lead to bias of the analysis results. For example, accidental correlations caused by individual differences among patients with IDD. Therefore, larger cohorts and more clinical data will be required in future studies, and validation should be conducted in patients of different age groups and with different imaging features to confirm the diagnostic value of these biomarkers in IDD. In addition, the specific molecular mechanism of *BID*, *KANK2*, and *SMIM3* in IDD has not been explored in the current study and pharmaceutical evaluation of these genes is lacking. For better clinical application, a great deal of in vivo and in vitro experiments is required to perform in the future. In vitro, overexpression or knockdown models of *BID*/*KANK2*/*SMIM3* can be constructed using primary NPCs from IDD patients and control groups to detect changes in NPC senescence, proliferation, apoptosis, and ECM metabolism. In vivo, IDD animal models can be established, and gene overexpression vectors or small-molecule regulators can be locally delivered to degenerated intervertebral discs. Intervertebral disc degeneration can be evaluated via MRI and histological staining, while immune cell infiltration and senescence phenotypes in disc tissues can be detected to clarify the in vivo roles of these genes. Finally, there are discrepancies in the infiltration of CD8^+^ T cells between MCP-counter and ssGSEA analyses. Specifically, MCP-counter primarily quantifies the total infiltration abundance of CD8^+^ T cells, while ssGSEA evaluates the enrichment level of effector memory CD8^+^ T cells. This discrepancy may be associated with immunophenotypic remodeling. However, further experiments such as flow cytometry are required to determine the specific changes in the proportions of various CD8^+^ T cell subsets.

## Conclusion

This current study identified 3 diagnostic biomarkers (*BID*, *KANK2*, and *SMIM3*) associated with senescence in IDD, which were mainly participated in the NF-kappa B, TNF, IL-17, and NOD-like receptor signaling pathways. Moreover, these biomarkers exhibited positive correlation with most immune cell infiltration. This study could provide promising diagnostic markers for IDD patients and lay a foundation for understanding the pathogenesis of IDD.

## Data Availability

The data sets generated and/or analyzed during the current study are available in the GSE70362 repository, [https://www.ncbi.nlm.nih.gov/geo/query/acc.cgi?acc=GSE70362], GSE15227 repository, [https://www.ncbi.nlm.nih.gov/geo/query/acc.cgi?acc=GSE15227] and GSE147383 repository, [https://www.ncbi.nlm.nih.gov/geo/query/acc.cgi?acc=GSE147383].

## Abbreviations

AUC: Area under the ROC curve
DAVID: Database for annotation, visualization and integrated discovery
DEGs: Differentially expressed genes
ECM: Extracellular matrix
NPC: Nucleus pulposus cell
SASP: Senescence-associated secretory phenotype
FC: Fold change
GEO: Gene Expression Omnibus
GO: Gene ontology
GSVA: Gene set variation analysis
IDD: Intervertebral disc degeneration
IL: Interleukins
KEGG: Kyoto encyclopedia of genes and genomes
LASSO: Least absolute shrinkage and selection operator
LBP: Lower back pain
MCP-Counter: Microenvironment cell populations-counter
MDSC: Myeloid-derived suppressor cell
MRI: Magnetic resonance imaging
NES: Normalized enrichment score
NF: Nuclear factor
OOB: Outofbag
ROC: Receiver operating characteristic
SRGs: Senescence-related genes
EMT: Epithelial–mesenchymal transition
ssGSEA: Single sample gene set enrichment analysis
1-TOM: 1 Topological overlap matrix
TNF: Tumor necrosis factor
WGCNA: Weighted gene correlation network analysis
